# Starting then stopping: a nationwide register-based study on the magnitude, predictors, and urban-rural patterns of under-vaccination variation across health centers in The Gambia

**DOI:** 10.1080/16549716.2024.2348788

**Published:** 2024-06-03

**Authors:** Alieu Sowe, Fredinah Namatovu, Bai Cham, Per E. Gustafsson

**Affiliations:** a Department of Epidemiology and Global Health, Umeå University, Umeå, Sweden; b Expanded Program on Immunization, Ministry of Health, Banjul, The Gambia; c Medical Research Council Unit The Gambia at the London School of Hygiene and Tropical Medicine, Bakau, The Gambia; d School of Public Health, Georgia State University, Atlanta, GA, USA

**Keywords:** Vaccination, coverage, under immunized, inequality, equity

## Abstract

**Objectives:**

Six million children were under-vaccinated in 2022. Our study aimed to 1) quantify the magnitude of under-vaccination variation between health facilities, 2) assess to which extent individual and health center level factors contributed to the variation, 3) identify individual and health facility factors associated with under-vaccination, and 4), explore rural vs. urban health facility variations.

**Methods:**

We used data from 61,839 children from The Gambia national routine vaccination register. We cross tabulated under-vaccination status across study variables and fitted two-level random intercept multilevel logistic regression models to measure variance, contribution to the variance, and factors associated with the variance and under-vaccination.

**Results:**

We found that 7% of the prevalence of under-vaccination was due to variation between health facilities. Thirty-seven percent of the variation was explained by individual and health center variables. The variables explained 36% of the variance in urban and 19% in rural areas. Children who were not vaccinated at 4 months or with delayed history, due for vaccination in the rainy season, and health facilities with very small or large population to health worker ratios had higher under-vaccination odds.

**Conclusion:**

Our study indicates that one of the pathways to improving vaccination coverage is addressing factors driving under-vaccination inequities between health facilities through urban-rural differentiated strategies.

## Paper context

- **Main findings:** Variation in under-vaccination rates between health centers contributes to worsening performance overall and in urban and rural areas.
- **Added knowledge:** Our study indicates that one of the paths to improving under-vaccination and consequently total coverage is by addressing the factors driving under-vaccination and its inequity between health facilities.
- **Global health impact for policy and action:** Children with delayed or non-vaccination history at four months of age, those due for vaccination in the rainy season especially in urban areas, and health facilities with very small or big birth dose to health worker ratios are potential targets for improving under-vaccination.

## Background

Despite the significant gains made in vaccination coverage, millions of children remain vulnerable to vaccine-preventable diseases due to under-vaccination globally [1,2]. Under-vaccination is the initiation of vaccination but failing to complete the schedule, commonly known as ‘dropout’ [3]. It presents as a decline in vaccination coverage as the recommended age for vaccination increases. Under-vaccination represents a systemic failure to consistently deliver initiated vaccinations to ensure completion of the schedule. It represents a critical challenge related to vaccination service utilization and exposes children to vaccine-preventable diseases [2,4]. Building strong immunization programs by achieving high life course vaccination coverage by 2030 as highlighted in the Immunization Agenda 2030 (IA2030) necessitates ensuring all children who had started vaccinations continue through to completion [5]. Achieving this goal requires implementing targeted strategies aiming to improve coverage including the reduction of under-vaccination.

Identifying factors driving under-vaccination is a necessary first step to design effective interventions against it. The determinants of under-vaccination are complex and operate at different nested levels. Applying an ecosocial theory perspective [6], these factors can be grouped into child-related, health facility related, and broader contextual aspects such as urban-rural residence. At the child (individual) level, several socio-demographic factors, including place of residence, delays in receipt of previous vaccine doses, internal migration, and season of the year, have been identified as contributors to the non-completion of scheduled vaccinations [7–13]. Moving to the health center level, previous research has also shown that caregivers could have preferences for the type of health facility (public vs private) to take their children for vaccination [14] and that vaccination rates could differ between health centers [13,15]. Yet, to our knowledge, the extent to which health facility-level heterogeneity contributes to dropping out from the vaccination schedule has not been previously studied. Additionally, it is common knowledge that health facilities require human resources to deliver vaccination services. However, studies investigating association between health worker density and vaccination coverage have been primarily conducted at higher levels of healthcare system, such as country and levels beyond health facilities [16–18]. This is in part due to lack of health facility-level health worker data. Health centers are the main points of contact for the delivery of vaccination services, and therefore, a readily intervenable target for vaccination improvement.

Under-vaccination is a bigger challenge to overall vaccination coverage than non-vaccination in countries with low absolute zero-dose children such as The Gambia, where zero-dose prevalence in surveys has usually been less than 1% [19,20]. The Gambia’s routine vaccination schedule for children is composed of 11 vaccines with a total of 23 recommended doses. The administration of the 23 doses is as follows: three doses at birth; four doses at two, three, and 4 months respectively after birth; one dose at 1 year; one dose at 16 months; and two doses at 18 months [21]. Receipt of all basic vaccine doses recommended in the first year of life is a commonly used indicator for full vaccination coverage within the immunization field. The last of the basic doses is recommended from 9 months of age in The Gambia. Coverage across the national schedule is marked by a prominent decline in coverage as the age of vaccination increases underscoring the challenge of maintaining consistent vaccination rates as children grow older [20,22]. All routine vaccines are provided to service providers free of charge by the national vaccination program. Vaccination demand generation activities to improve performance such as advocacy, communication, and social mobilization which are routinely implemented. Vaccinated children are issued infant welfare cards which is a booklet for recording the child’s biodata, vaccine doses administered, deworming and vitamin A supplementation, weight, and health facility visits for curative services.

Reducing vaccination coverage disparities among social groups is recognized as beneficial for disadvantaged groups and overall coverage improvement. This recognition has prompted the identification of several dimensions of inequity for monitoring vaccination coverage [23]. Urban-rural coverage inequity is well highlighted in The Gambia through multiple household surveys [19,20,22,24], highlighting the importance of broader geographical contexts for vaccination coverage. The observed variation in coverage based on urban-rural residence implies that variations in under-vaccination at health facility level, and the factors influencing these variations, may differ between urban and rural areas necessitating monitoring urban-rural inequality in The Gambia.

We aimed to address four objectives with a view of generating evidence that could inform targeted interventions by the vaccination program to effectively address under-vaccination. Firstly, to measure the magnitude of variation in under-vaccination between health facilities. Secondly, to assess to which degree individual and health center factors contributed to the variation between facilities. Thirdly, identify specific individual and health facility level factors associated with under-vaccination. Finally, we aimed to explore whether patterns of under-vaccination variation and determinants are similar among rural and urban health facilities.

## Methods

### Data source

This study utilized data from The Gambia national vaccination register. The register is a hybrid of smart paper and digital components. The registration process involves the recording of the details of each vaccine recipient, including their biodata and vaccination history, on smart paper forms which have predesigned fields where health workers write details of beneficiaries. Subsequently, these forms are then scanned and transformed into digital records by the smart paper engine. A pilot phase of the smart paper system took place in two of the seven health regions in the country in 2017, and it was officially rolled out nationwide in January 2021 [25].

Our study focused on the age cohort 12–23 months who had received at least one birth dose vaccine and at least one 2-month vaccine dose with vaccination data recorded between January 2021 and December 2022. This choice was based on three reasons. First, the age cohort 12–23 months is recommended for assessing vaccine doses administered during the first year of a child’s life as in our case [26]. Second, our 2-month vaccine dose cutoff point aligns with the global operational definition of zero-dose children for the IA2030. This definition considers children who have not received the first dose of a diphtheria-pertussis-tetanus containing vaccine, administered at 2 months in The Gambian context, as zero-dose [27]. Finally, the period selected is ideal considering the national implementation of the electronic register and data verification processes. The final dataset in our analysis comprised of data of 61,839 children from 80 health facilities across the country. We excluded 16,066 children who did not meet the inclusion criteria.

### Measures

The variables used in this study were selected based on their availability in the dataset or the possibility to operationalize them from the available data.

#### Outcome variable

Under-vaccination was based on 9-month vaccine doses and operationalized as follows: a child was considered vaccinated if he/she received at least one of the four vaccines recommended for administration at 9 months (coded as ‘1’), and not vaccinated if the child had not received any of the four doses (coded as ‘0’). This definition is a modified version of the standard way of calculating dropout rates where a single later dose is compared with an earlier dose [3]. Our previous work has shown that a child can miss opportunities for simultaneous vaccination (MOSV) meaning that he/she can receive some but not all due doses in the same vaccination session [28]. Considering the possibility of MOSVs and our intention to ascertain whether a child came in contact with the vaccination system when their recommended 9 month doses were due, receipt of at least one of the doses recommended at 9 months is sufficient to prove a child had returned for vaccination.

We chose 9-month doses for three reasons. First, dropout rates for 9-month vaccines are notably higher than those for earlier doses, making this set of doses an important intervention point [20,22]. Second, the measles vaccine which happens to be the IA2030s core tracer indicator for identifying weaknesses in immunization programs and guide vaccination program planning [5] is administered at nine and 18 months in The Gambia. Achieving a minimum coverage of 95% for both the 9-month and 18-month doses is a crucial milestone for countries in the African region to be considered on track for measles elimination [29]. Unfortunately, The Gambia has faced challenges in reaching and maintaining such high vaccination coverage rates, resulting in periodic measles outbreaks and stalling progress toward measles elimination [19,20,22,30–32]. Finally, the fourth dose of the polio vaccine and the first and only dose of the yellow fever vaccine, all of which have outbreak potential, are administered at 9 months [33,34].

#### Individual level variables

Individual level variables included sex of the child, status of fourth month vaccines, number of health facilities the child received vaccines from, and the quarter of the year the vaccines were due. These variables were operationalized as follows; the sex of a child was either male or female while the status of fourth month doses was categorized into three groups: ‘vaccinated on time’, ‘not vaccinated’ or ‘delayed vaccination’. We grouped children into ‘one health facility’ for those who received vaccines from a single facility and ‘at least two health facilities’ for those who received vaccines from more than one. The quarter of the year the vaccine doses were due was categorized ‘first’, ‘second’, ‘third’, and ‘fourth’. We chose quarters of the year instead of categorizing them into wet and dry season to provide a more nuanced analysis. The rainy season runs from around mid-June to around mid-October in The Gambia with August being the rainiest month. The harvesting of farms produces is from October to December.

#### Health facility level variables

At the health facility level, vaccination session type, health facility type, and birth dose to health worker ratio were the variables used. *Vaccination sessions* were considered ‘static’ or ‘outreach’ depending on where they were conducted in The Gambia. Static sessions are sessions that are conducted in a health center with a fridge for storing vaccines and health staff for vaccine administration. An outreach session, on the other hand, is a vaccination session in which a team with vaccine carriers or cold boxes moves from one health center to another or a community on a regular monthly basis. *Health facility type* was either ‘public’ (government owned) or ‘private’ (all other ownerships). *Birth dose to health worker ratio* was derived from two variables. First, we constructed a variable named ‘birth doses’ with a value of ‘1’ if a child received any of the three birth doses in the national schedule and ‘0’ if the child received none of them. Then we summed the birth doses by facility and divided the result by the average number of public health officers posted to that health facility during the study period to obtain the birth dose to health worker ratio. Public health officers are a dedicated cadre of health workers who administer routine vaccines in The Gambia. The resulting ratios for various health facilities were then recategorized into three groups: ‘less than 100’, ‘100–299’, and ‘300 & above’. Data on human resource availability in health facilities for vaccination was obtained from regional health directorates in charge of health workers in their respective regions.

### Urban-rural stratification variable

Urban-rural residence is an important vaccination inequity dimension in The Gambia, but it is not recorded in the national vaccination register. We therefore used the information on vaccination session type (static vs outreach) as a proxy for place of residence (urban vs rural), as in The Gambia, almost all urban settlements are served by static vaccination sessions while rural settlements are mainly served by outreach vaccination sites. To maintain consistency across the manuscript, static vaccination sessions are referred to as urban and outreach sessions as rural henceforth.

### Data analysis approach

#### Descriptive statistics

We cross tabulated under-vaccination status (categorized as vaccinated vs. not vaccinated) and each of individual and health facility level variables and reported frequencies and Chi Squared P-values. We also reported distribution of children across categories of variables.

#### Main analysis

We used a two-level random intercept multilevel logistic regression modelling approach using Stata 18 and fitted two models to answer the four aims of the study. An empty multilevel model (no exposure variable) with health facilities as the second level and 9-month vaccination status as the outcome was first fitted (Model 1). This model was used to estimate the magnitude of health center variance in under-vaccination (aim 1). While the variation was relatively small, it justified the use of multilevel modeling to account for the clustering effect within facilities [35]. Following that, we fitted the second model (Model 2) by integrating both individual and health center variables to assess the proportion of the inter-health facility variance explained by individual and health center level variables in our study (aim 2), as compared to the variance presented in Model 1. We also used Model 2 to identify the individual and health facility factors associated with under-vaccination (aim 3). To examine rural–urban patterns in factors explaining under-vaccination variation between health centers and in factors associated with under-vaccination (aim 4), we refitted an empty (Model 1) and a fully adjusted model (Model 2) stratified by urban-rural residence as was done for aims 1 and 2.

Random effects of each model were reported as health facility variance, intra-cluster correlation coefficient (ICC), median odds ratio (MOR), and proportional change in variance (PCV). Fixed effects were presented as adjusted odds ratios (aORs) and their corresponding 95% confidence intervals (CI). We evaluated model performance using the Akaike Information Criterion (AIC) model fit statistic, a better fit model is expected to have a smaller AIC value. We plotted caterpillar plots to show the ranking of health facilities, in overall sample and by urban-rural area, based on their under-vaccination likelihood using the logarithm values of their odds ratios. An odds ratio of 1 indicating no difference corresponds to 0 log odds ratio in the logarithm scale [36].

To assess multicollinearity, we calculated variance inflation factors (VIFs) for the independent variables. The highest VIF was for vaccination session type, at 1.09, suggesting that collinearity may not be a substantial problem in our analysis.

## Results

### Descriptive statistics

Table 1 presents the frequency of under-vaccination across individual and health center variables. Among individual level variables, children who had not receive their four-month vaccines, those who received vaccinations from one health center, and those who were due for vaccination in the third quarter of a year had the higher frequencies of under-vaccination, with rates of 56.4%, 20.1% and 19.1% respectively. There was no difference in under-vaccination between male and female children. For health facility level variables, private health facilities (19.8%) and a birth dose to health worker ratio of at least three hundred (20.2%) had larger proportions of under-vaccination in their respective categories.

**Table 1.** Prevalence of under-vaccination (9-month doses) by study variables among 12–23 months old children in The Gambia, in the total sample and by rural-urban health facility.

Variable (*N* = 61839)	Overall total			Rural			Urban		
	Total N (%)	Under-vaccinated N (%)	χ2 *p*–value	Total N (%)	Under-vaccinated N (%)	χ2 *p*–value	Total N (%)	Under-vaccinated N (%)	χ2 *p*–value
**Total**	**61,839 (100.0)**	**11,160 (18.0)**	<0.001	**18,427 (29.8)**	**2,623 (14.2)**		**43,412 (70.2)**	**8,537 (19.7)**	
**INDIVIDUAL FACTORS**									
***Sex***			0.708			0.902			0.752
Female	30,387 (49.1%)	5,466 (18.0)		9,090 (49.3%)	1291 (14.2)		21,297 (49.1%)	4,175 (19.6)	
Male	31,452 (50.9%)	5,694 (18.1)		9,337 (50.7%)	1332 (14.3)		22,115 (50.9%)	4,362 (19.7)	
***Status of 4-month vaccines***			<0.001			<0.001			<0.001
Not vaccinated	7,227 (11.7%)	4,076 (56.4)		1,835 (10.0%)	816 (44.5)		5,392 (12.4%)	3,260 (60.5)	
Vaccinated on time	43,371 (70.1%)	5,382 (12.4)		13,754 (74.6%)	1379 (10.0)		29,617 (68.2%)	4,003 (13.5)	
Delayed vaccination	11,241 (18.2%)	1,702 (15.1)		2,838 (15.4%)	428 (15.1)		8,403 (19.4%)	1,274 (15.2)	
***Number of health facilities visited***			<0.001			<0.001			<0.001
One	40,365 (65.3%)	8,099 (20.1)		12,798 (69.5%)	1999 (15.6)		27,567 (63.5%)	6,100 (22.2)	
At least two	21,474 (34.7%)	3,061 (14.3)		5,629 (30.5%)	624 (11.1)		15,845 (36.5%)	2437 (15.4)	
***Quarter of year 9-month vaccines due***			<0.001			0.010			<0.001
First	11,655 (18.8%)	2,041 (17.5)		3,933 (21.3%)	600 (15.3)		7,722 (17.8%)	1441 (18.7)	
Second	13,479 (21.8%)	2,433 (18.1)		4,008 (21.8%)	543 (13.6)		9,471 (21.8%)	1890 (20.0)	
Third	20,712 (33.5%)	3,952 (19.1)		5,601 (30.4%)	835 (14.9)		15,111 (34.8%)	3117 (20.6)	
Fourth	15,993 (25.9%)	2,734 (17.1)		4,885 (26.5%)	645 (13.2)		11,108 (25.6%)	2089 (18.8)	
**HEALTH FACILITY FACTORS**									
***Health facility type***			<0.001			0.168			0.770
Private	5,755 (9.3%)	1,141 (19.8)		20 (0.1%)	5 (25.0)		5,735 (13.2%)	1136 (19.8)	
Public	56,084 (90.7%)	10,019 (17.9)		18,407 (99.9%)	2618 (14.2)		37,677 (86.8%)	7401 (19.6)	
***Birth dose to health worker ratio***			<0.001			<0.001			<0.001
Less than 100	3,352 (5.4%)	656 (19.6)		867 (4.7%)	92 (10.6)		2,485 (5.7%)	564 (22.7)	
100–299	33,163 (53.6%)	5,396 (16.3)		13,425 (72.9%)	1863 (13.9)		19,738 (45.5%)	3533 (17.9)	
300 & above	25,324 (41.0%)	5,108 (20.2)		4,135 (22.4%)	668 (16.2)		21,189 (48.8%)	4440 (21.0)	

Regarding urban-rural patterns, the urban health facilities had a higher frequency of under-vaccination, at 19.7%. The frequencies of individual variables and under-vaccination in both the rural and urban strata were generally consistent with those observed in the overall sample with the exception of the health facility type. Private health center ownership was uncommon in rural areas, hence the low absolute number of under-vaccinated children. Surprisingly, private health facilities had higher frequency of under-vaccination in the overall sample but there was no statistically significant difference in the urban-rural stratified analysis.

### Magnitude of variation in under vaccination between health facilities and the contribution of individual and health center level factors to this variation

**Figure 1.**
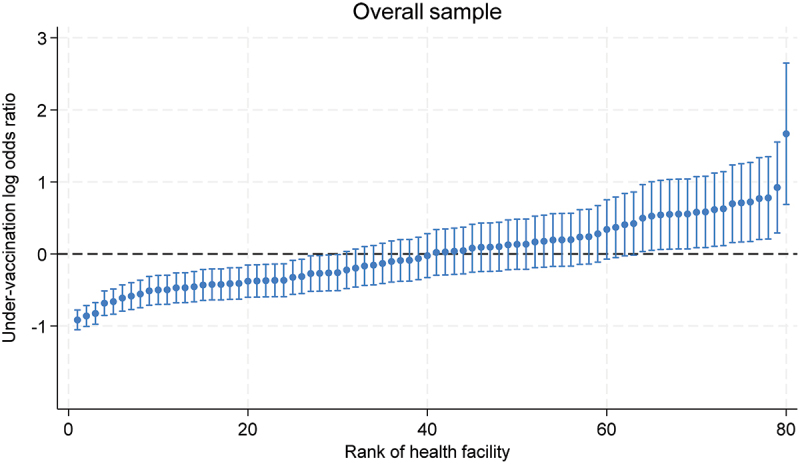
Health facility differences in under-vaccination risk in the overall sample obtained from a null multilevel regression model.

A ranking of health facilities by under-vaccination risk is presented in Figure 1. As can be observed in Figure 1, there was a significant variation in the log odds ratio of under-vaccination across health centers, ranging from −1.05 to 2.65 in the overall sample, corresponding to an under-vaccination prevalence of 6.1% in the best-ranked health center to 57.3% in the worst-ranked health facility. Table 2 shows the results of two multilevel logistic regression models. The random effects observed in the null model (Model 1) indicated a between health center ICC of 0.07 highlighting that, 7% of the inter-individual variation in under-vaccination is explained by heterogeneity between health centers. The MOR of 1.62 in the first model implies that, on average, there is 62% increased odds of under-vaccination moving from a facility with a lower to one with a higher under-vaccination rate.

**Table 2.** Multilevel logistic regression analysis of the association of child level and health facility level variables and under-vaccination among children 12–23 months in December 2022 in The Gambia.

Measure	Model 1	Model 2
	Estimate (95% CI)	Estimate (95% CI)
**FIXED EFFECTS**		
***Sex***		
Female		reference
Male		1.00 (0.95–1.04)
***Status of fourth month vaccines***		
Vaccinated on time		reference
Not vaccinated		8.30 (7.85–8.77)
Delayed vaccination		1.13 (1.06–1.20)
***Number of health facilities child received vaccines***		
One		reference
At least two		0.64 (0.60–0.67)
***Quarter of year nineth month vaccines due***		
First		reference
Second		1.01 (0.94–1.08)
Third		1.08 (1.01–1.15)
Fourth		0.97 (0.91–1.04)
***Vaccination session type***		
Rural		reference
Urban		1.06 (0.99–1.13)
***Health facility type***		
Public		reference
Private		1.32 (1.02–1.70)
***Birth dose administration to health worker ratio***		
Less than 100		reference
100–299		0.65 (0.49–0.87)
300 & above		0.87 (0.65–1.18)
**RANDOM EFFECTS**		
Health facility variance	0.258 (0.182–0.366)	0.163 (0.112–0.236)
ICC*	0.073 (0.052–0.100)	0.047 (0.033–0.067)
PCV**	reference	36.89%
MOR**3	1.623	1.469
Model fit statistics		
Log likelihood	−28556.43	−25411.25
AIC****	57116.86	50849.15

*ICC means intra-cluster correlation coefficient.

**PCV means Proportional Change in Variance.

***MOR means Median Odds Ratio.

****AIC means Akaike Information Criterion.

Upon introducing individual and health facility level variables (Model 2), a total of 37% (PCV) of the variance between health facilities was explained. As auxiliary analysis, two intermediate models were run: one with only individual variables and the other with health facility variables (see Appendix). The results of the auxiliary analysis showed that about 28% points of the 37% explained variation in dropout rates between health centers was attributed to differences in the distribution of health facility variables and the remaining 9% points were accounted for by differences in individual-level characteristics. Model 2 was a better fit model based on its smaller AIC.

### Individual and health facility factors associated with under-vaccination

For health center variables of the fixed effects in Model 2 of Table 2, differences can be observed according to facility type and by birth dose to health worker ratio. Children who received vaccinations in private centers had higher odds (aOR = 1.32) of under-vaccination compared to their counterparts vaccinated in public facilities. Children who were vaccinated in health centers with a birth dose to health worker ratio of 100–299 showed lower odds of under-vaccination than those vaccinated in facilities with a ratio of less than 100. Surprisingly, children from health facilities with a high birth dose to health worker ratios (300 & above) were not statistically different in under-vaccination in comparison with those from health facilities with a ratio of less than 100.

Focusing on the individual level factors, the sex of a child was the only variable not associated with under-vaccination. In terms of the vaccination status for four-month vaccines, prominent higher odds of under-vaccination was found among children who were not vaccinated at all for four-month doses (aOR = 8.30) and children who experienced delays in their vaccination (aOR = 1.13), compared to their counterparts who were vaccinated on time. Furthermore, children who were taken to more than one health center for vaccination exhibited lower odds (aOR = 0.64) of missing their vaccines compared to those who received vaccines at a single facility. Considering the timing of 9-month vaccines, with the first quarter of the year as the reference category, children due to receive their vaccines in the third quarter had higher odds (aOR = 1.08) of not receiving them.

### Patterns in rural and urban areas in factors explaining under-vaccination variation between health facilities and in factors associated with under-vaccination

**Figure 2.**
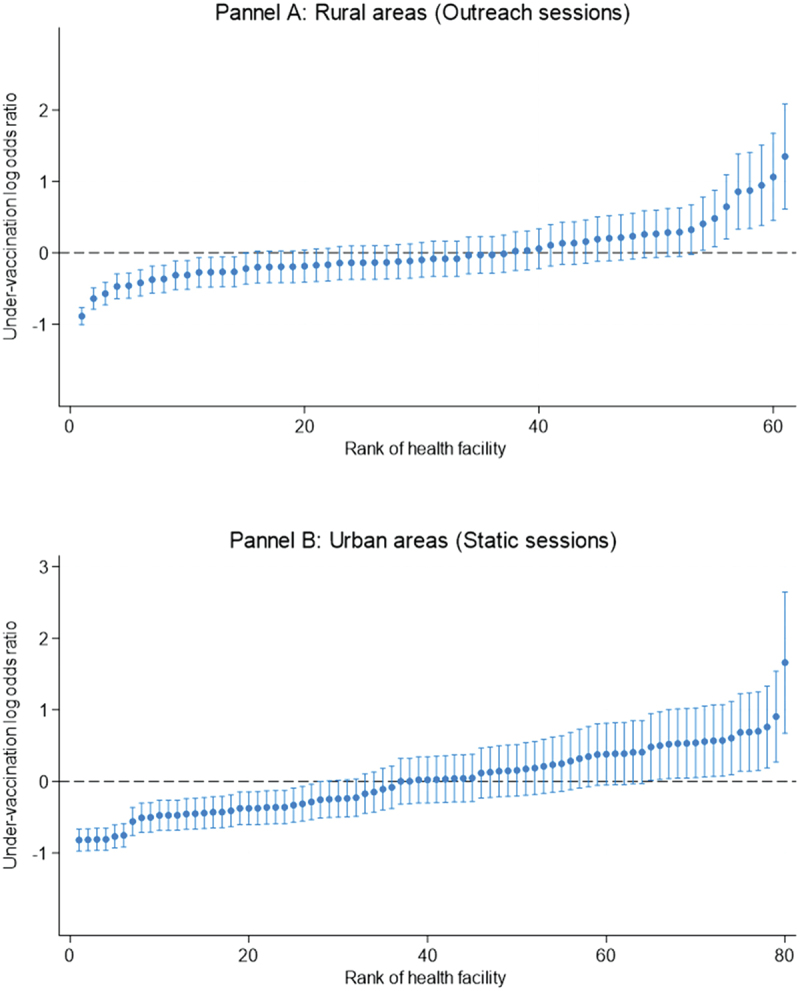
Health facility differences in under-vaccination risk in urban and rural areas obtained from null multilevel regressions.

The urban-rural stratified results presented in Table 3 displays a noticeably different pattern than those in the overall analysis in Table 2. Intracluster correlation in rural areas was slightly (12%) higher than that of rural areas (ICC = 0.064 in rural and 0.075 in rural) and similar to the total sample analyses (ICC = 0.073). There was a substantial difference in the proportions of variance explained by the study variables in urban vs rural strata. In the rural setting, only 19% of the variance was accounted for by the variables while in the urban stratum, they explained 36% of the variance, similar to the analysis of the total sample. Under-vaccination log odds ratios (Figure 1) from the best to the worst performing health facility ranged from −1.00 to 2.09 (Panel A) in rural areas and from −0.97 to 2.65 (Panel B) in urban areas. In terms of under-vaccination prevalence in the best and worst performing health centers, the log odds correspond to 0%−39% and 3.9%−57% in rural and urban areas respectively (Figure 2).

**Table 3.** Multilevel logistic regression analysis of the association of individual and health facility level variables and under-vaccination among children 12–23 months stratified by rural–urban residence in The Gambia, 2022.

Measure	Rural		Urban	
	Null model	Adjusted Model	Null model	Adjusted Model
**FIXED EFFECTS**				
***Sex***				
Female		reference		reference
Male		0.99 (0.90–1.08)		1.00 (0.95–1.05)
***Status of fourth month vaccines***				
Vaccinated on time		reference		reference
Not vaccinated		6.47 (5.78–7.23)		9.06 (8.49–9.68)
Delayed vaccination		1.48 (1.31–1.67)		1.04 (0.96–1.11)
***Number of health facilities child received vaccines***				
One		reference		reference
At least two		0.63 (0.56–0.70)		0.63 (0.60–0.67)
***Quarter of year nineth month vaccines due***				
First		reference		reference
Second		0.81 (0.71–0.92)		1.10 (1.01–1.20)
Third		0.89 (0.79–1.01)		1.17 (1.08–1.26)
Fourth		0.82 (0.72–0.94)		1.03 (0.95–1.12)
***Health facility type***				
Public		reference		reference
Private		1.98 (0.58–6.81)		1.28 (0.99–1.66)
***Birth dose administration to health worker ratio***				
Less than 100		reference		reference
100–299		1.21 (0.73–2.02)		0.59 (0.44–0.80)
300 & above		1.69 (0.95–3.01)		0.81 (0.59–1.1)
**RANDOM EFFECTS**				
Variance	0.226 (0.146–0.350)	0.183 (0.116–0.287)	0.268 (0.184–0.391)	0.171 (0.115–0.256)
ICC*	0.064 (0.042–0.096)	0.053 (0.034–0.080)	0.075 (0.053–0.106)	0.049 (0.034–0.072)
PCV**	Reference	19.027%	Reference	36.194%
MOR***	1.57358	1.50332	1.63819	1.48411
AIC****	14693.12	13555.47	42392.17	37211.58

*ICC means intra-cluster correlation coefficient.

**PCV means Proportional Change in Variance.

***MOR means Median Odds Ratio.

****AIC means Akaike Information Criterion.

Dissimilarities were evident in three variables: birth dose to health worker ratio, the status of four-month vaccines, and the quarter of the year when nine-month vaccines were due. At the health center level, birth dose to health worker ratio was not associated with the odds of under-vaccination in rural areas, but a birth dose to health worker ratio of 100–299 was associated with 41% lower under-vaccination odds (adjusted odds ratio = 0.59) compared with ratio of less 100 in urban areas. The association between health center type and under-vaccination disappeared in the urban-rural stratified analysis. At individual level, non-vaccination and delayed vaccination at four months were associated with under-vaccination in rural areas, but this association was observed only for non-vaccination in urban areas. Regarding the due quarter for nine-month vaccines, in rural areas it can be observed that the second and fourth quarters of the year had lower odds of under-vaccination compared to the first quarter whereas the third quarter manifested higher odds at the individual level in urban areas.

## Discussion

Our study aimed quantify the magnitude of variation in under-vaccination between-health facilities, assess the extent to which individual and health facility level factors contributed to the variation and their association with under-vaccination, and describe the corresponding patterns by urban and rural areas. First, our analysis showed a modest but significant variation in the distribution of under-vaccination between health facilities, indicating that some health facilities performed relatively better than others in continuing with children along the vaccinations schedule. Second, our findings highlighted that individual and health center factors were associated with between health facility variation in under-vaccination. Third, individual and health center factors were associated with the odds of under-vaccination. Lastly, urban-rural patterns in inter-health center under-vaccination variation were similar but the association between some individual and health facility factors and dropping out differed between rural and urban areas. Taken together, these findings underscore variation in under-vaccination across health facilities, its association with both child-level and health facility-level factors, and urban-rural differences in factors associated with the variation.

Earlier studies assessing differences in vaccination between health centers mainly used health center or health center group level measures to show differences between health facilities [15,37]. Our study contributes to the literature on vaccination coverage by adding a perspective on heterogeneity at the health center-level and its role in under-vaccination using real-world data from a nationally implemented routine vaccination registry. The small but significant variation in under-vaccination observed in our study suggests under-vaccination is more of a problem across all health facilities than it is heterogeneity problem. The fact that just over one-third of the total health facility variation could be explained by the individual (8% points) and facility (29% points) factors in our study indicates that there are other factors contributing to the observed health facility variance beyond those investigated in our study. Identifying factors accounting for the unexplained difference between health facilities is one of the potential areas for future research in understanding health center level inequity in under-vaccination. These factors could potentially include health center infrastructure, the quality of vaccination service delivery, and factors related to the population served such as sociodemographic characteristics [7,15], data on which was unavailable for our study.

Focusing on health facility level variables, we observed a higher prevalence of under-vaccination in private health centers compared to public health centers. The difference between public and private health centers could possibly in part be explained by the service fee levied by some private facilities. Vaccination fees have been identified as one of the barriers to vaccination [38]. Generally, vaccination is free in The Gambia and no public health center charges a fee, however, some private health centers do charge fees for vaccination services. For birth dose to health worker ratio, we identified an interesting pattern in its relationship with under-vaccination. We found health facilities that were in the middle of the three categories were in the most advantageous position compared to those in the group with the smallest ratio in the overall analysis but only in urban areas in the stratified analysis. Our findings contradict those of some previous studies comparing vaccination coverage with human resources, which concluded that increasing the number of human resources for vaccination would potentially lead to higher coverage [16,18]. One potential explanation of the differences between our study and the other studies could be the differences in methodology. Our study estimated density at the service delivery level, specifically the health facility, while the other studies did so at higher administrative levels. However, it is also possible that our observations were confounded, in the sense that some health workers in the two most urban regions (home to about 60% of the population) might be concurrently pursuing academic programs at higher education institutions while working since most higher education institutions are located in those two regions. This is especially plausible when considering that the association remained only in urban areas after stratification. An earlier study conducted in The Gambia reported that urban caregivers experienced longer waiting times and more crowded vaccination sessions than rural carers [39]. This may be a factor that can potentially discourage mothers especially in urban areas from subsequent vaccinations. Another potential factor could be avoidance of vaccine wastage by batching children. For example, a health worker may decide not to open a measles containing vaccine vial for just one or two children and discard the rest even though the national program emphasizes that the vial should be opened even if there is just one child. However, we addressed this in our operationalization by considering administration of at least one of the three vaccine doses administered at nine months. One of the doses is the fourth dose of the oral polio vaccine which can actually be reused for up to one month provided the recommended criteria are met. Therefore, a child will not be rescheduled for vaccination for fear of vaccine wastage. Nevertheless, our finding underscores the general principle that deployment of human resources should consider expected workload at the place of work to maximize efficiency.

The finding of delays in or non-receipt of four-month vaccines being negatively associated with under-vaccination is consistent with other findings in the literature [8,9]. This observation highlights that signs of future under-vaccination could be manifested early on in the vaccination schedule. Therefore, in spite of the under-vaccination gap being more visible from nine-month vaccines, signals such as delayed and non-receipt of earlier doses are early cues for action to prevent larger under-vaccination proportions for later doses in the schedule. Children who received vaccination services in more than one facility were less prone to under-vaccination. This could be partly attributed to parental/caregiver knowledge on vaccination such as the schedule, the importance of vaccination, and knowledge of the availability of services outside the caregivers usual residence. Caregivers who value vaccination and are aware that their children can receive vaccines where they are travelling to will likely take their children’s vaccination cards with them when travelling to have them vaccinated. At the individual level, our study reinforced that the sex of a child is not a significant determinant of their vaccination status in The Gambia [40,41], signifying a positive attitude that parents likely do not differentiate between male and female children when seeking for preventive health services.

In the overall sample, we observed that children who were due for vaccination in the third quarter of the year, corresponding to the period July to September, are more likely to be under-vaccinated than their counterparts who were due to receive their vaccines in the first quarter of a year (January to March). This may be explained by the third quarter of the year being the core of the rainy season which normally runs through June to October [42]. Similar to ours, a study in Madagascar also reported lower vaccination rates during the rainy season [12]. The rainy season also comes with mobility challenges such as muddy roads and stagnant water, especially in urban areas, which could potentially deter caregivers in urban areas from taking their children for vaccination services. Since majority of Gambians are farmers, some parents and caregivers may be tempted to prioritize farming activities during the short rainy season period over taking their children for vaccination. Rural women prepare and go together groups to have their children vaccinated. The impact of the raining season on defaulting for vaccinations may have been buffered by stronger social networks in rural areas which most rural women mentioned as encouraging vaccination session attendance [39].

Surprisingly, our analysis did not show a difference in the odds of under-vaccination between rural and urban areas. The absence of a difference between urban and rural areas can be attributed to The Gambia’s well-established vaccination support structures for regular outreach vaccination [43]. It could also be taken to mean that under-vaccination is a general challenge across health facilities in both areas. While children due for vaccination in the third quarter of the year had higher odds of under-vaccination in the total sample and urban strata relative to the first quarter, this was not the case in rural areas where children due in the second and fourth quarters had lower odds. The finding that children with due vaccination dates in the second and fourth quarters of a year had lower odds of under-vaccination than those due in the first quarter (which in itself was similar to the third quarter) is an indication that the third quarter is also a potential target for improving under-vaccination in rural areas too. In relation to the magnitude of the contribution of variables in our study to variation in vaccination between health centers in urban vs rural areas, our results showed that the variables explained about twice the amount in urban areas than they did in rural areas. This suggests that factors contribute differently to inequity in under-vaccination between health centers.

### Methodological considerations

The main strength of our study is that the vaccination register covers all health facilities offering vaccination services in the country. This presents several practical advantages. First, the large sample size enhances statistical power, which is desirable. Second, the use of a routine register allows us to draw upon real-world evidence, a data source, which is familiar to policy makers at the program level and therefore promoting improvement action. This aspect was particularly important in the context of our study, where we focused on children who had interacted with vaccination services but subsequently dropped out of the vaccination schedule meaning that majority of the children are within reach of the vaccination system. Therefore, they can be targeted for vaccination. Third, it simplifies the monitoring of intervention impact since there is no need for specialized data collection. This too could promote the implementation and monitoring of interventions to improve under-vaccination in the Gambia and in other countries with similar settings.

Aside from the strengths mentioned above, we would like to highlight some limitations of this study. Vaccination registers as administrative data sources are created to collect minimal data required for program monitoring and reporting. Therefore, they are limited in variables. For example, demographic, economic, geographic, and health system factors related to service delivery are known to influence vaccination dropout, but these are not collected by the register we used. This might be a reason why the included variables explained just over a third of the observed variance among the health centers in the overall sample. The Gambia has introduced an electronic civil and vital statistics registration system (CRVS). Linking the CRVS to vaccination register in the future could help address this limitation in future. Our results appear to have shown a higher prevalence of under-vaccination than reported in previous surveys, such as the 2019/2020 demographic and health survey conducted in The Gambia [20]. This difference is potentially due to differences in methods. Surveys such as the DHS, collect vaccination history from mothers or caregivers of children who spent the previous night in surveyed households while our study used all age-eligible children in a national registry. Even though to a likely small extent, our approach can result in us not been able account for cases where children initiated vaccination in The Gambia but travelled out of the country before completing the vaccination schedule since we considered all children in the register. The time taken by health workers to verify vaccination data that was not recognized by the smart paper engine before it is updated in the vaccination register could also increase the number of children who may appear to have dropped out. However, we considered this by making an allowance of 4 months after December 2022, before obtaining data for the study. Therefore, this is unlikely to be an issue in our study. Investigating why children drop out is a potential area for future research.

We used vaccination session type as a proxy for urban-rural area stratification because specific enumeration area data was not available in the dataset we used. Using vaccination as a proxy is a reasonable choice given the distribution of session type by rural–urban residence in The Gambia. It is important to note that this proxy does not perfectly classify urban and rural areas. For example, some rural settlements in the country are served by static vaccination sessions. There are also some urban static sessions that serve rural residents introducing the possibility of misclassifying the urban-rural residence of children based on their vaccination session type. The findings on urban-rural residence should be interpreted with caution.

## Conclusions

Our study has shown that only approximately 7% of the variation of under-vaccination in The Gambia was due to differences between health facilities, which suggests a ubiquitous rather than localized problem of under-vaccination. Moreover, while about a third of this clustering was explained by individual and health facility factors in the total sample, the same factors were of double importance in urban areas, which suggest differentiated interventions in rural and urban areas to maximize their impact. Overall, children with delayed or non-vaccination history at 4 months of age, those due for vaccination in the rainy season especially in urban areas, and health facilities with very small or birth dose to health worker ratios are potential targets for improving under-vaccination. In conclusion, our study suggests that one of the paths to improving under-vaccination and consequently total coverage is by addressing the factors driving under-vaccination and its inequity between health facilities.

## Acknowledgments

Thanks to the Erling-Persson Foundation for funding Alieu’s PhD, of which this paper is a part. The Department of Epidemiology and Global Health of Umeå University facilitated the open access publication of this article. The Ministry of Health of The Gambia and Shifo Foundation also supported Alieu’s PhD through his continued employment with the Ministry of Health and Shifo fellowship. We thank the DHS program for providing the data used in this paper. Funding

## Appendix Appendix

### Auxiliary analysis

Multilevel logistic regression analysis of the association of child level and health facility level variables and under-vaccination among children 12 – 23 months in The Gambia.

**Table:**

	Model 1	Model 2	Model 3	Model 4
Measure	Estimate (95% CI)	Estimate (95% CI)	Estimate (95% CI)	Estimate (95% CI)
**FIXED EFFECTS**				
***Sex***				
Female		reference		reference
Male		1.00 (0.95–1.04)		1.00 (0.95–1.04)
***Status of fourth month vaccines***				
Vaccinated on time		reference		reference
Not vaccinated		8.30 (7.85–8.77)		8.30 (7.85–8.77)
Delayed vaccination		1.13 (1.06–1.20)		1.13 (1.06–1.20)
***Number of health facilities child received vaccines***				
One		reference		reference
At least two		0.64 (0.60–0.67)		0.64 (0.60–0.67)
***Quarter of year nineth month vaccines due***				
First		reference		reference
Second		1.01 (0.94–1.09)		1.01 (0.94–1.08)
Third		1.08 (1.01–1.16)		1.08 (1.01–1.15)
Fourth		0.97 (0.91–1.04)		0.97 (0.91–1.04)
***Vaccination session type***				
Rural			reference	reference
Urban			1.08 (1.02–1.15)	1.06 (0.99–1.13)
***Health facility type***				
Public			reference	reference
Private			1.27 (0.97–1.65)	1.32 (1.02–1.70)
***Birth dose administration to health worker ratio***				
Less than 100			reference	reference
100–299			0.65 (0.49–0.88)	0.65 (0.49–0.87)
300 & above			0.89 (0.65–1.22)	0.87 (0.65–1.18)
**RANDOM EFFECTS**				
Health facility variance	0.258 (0.182–0.366)	0.235 (0.164–0.337)	0.186 (0.129–0.264)	0.163 (0.112–0.236)
ICC*	0.073 (0.052–0.100)	0.067 (0.048–0.093)	0.054 (0.038–0.075)	0.047 (0.033–0.067)
PCV**	reference	8.75%	27.84%	36.89%
MOR**3	1.623	1.589	1.507	1.469
Model fit statistics				
Log likelihood	−28556.43	−25424.90	−28542.62	−25411.25
AIC****	57116.86	50867.80	57097.87	50849.15

*ICC means intra-cluster correlation coefficient.

**PCV means Proportional Change in Variance.

***MOR means Median Odds Ratio.

****AIC means Akaike Information Criterion.
